# A case of facial atrophic sarcoidosis in an adolescent, successfully treated with the combination of prednisone and hydroxychloroquine^☆^^☆☆^

**DOI:** 10.1016/j.abd.2019.08.028

**Published:** 2020-03-19

**Authors:** Xiaomei Zhu, Jianfang Sun

**Affiliations:** Department of Pathology, Institute of Dermatology, Chinese Academy of Medical Sciences, Peking Union Medical College, Nanjing, China

**Keywords:** Adolescent, Atrophy, Hydroxychloroquine, Prednisone, Sarcoidosis

## Abstract

Sarcoidosis is a multisystem granulomatous disorder of unknown aetiology. Cutaneous involvement occurs in up to 30% of patients and skin findings are often the initial presenting symptom. The facial atrophic form of sarcoidosis without associated ulceration in adolescents has rarely been described in the literature. We report a case of 13-year-old male patient with a facial atrophic sarcoidosis who was successfully treated with the combination of prednisone and hydroxychloroquine.

## Introduction

Sarcoidosis is a common systemic, noncaseating granulomatous disease of unknown aetiology. Cutaneous sarcoidosis, the “great imitator”, can baffle clinicians because of its diverse manifestations and its ability to resemble both common and rare cutaneous diseases. Morphologically, plaques and papules are the most commonly observed cutaneous lesions.^1^ Rare presentations including psoriasiform, ichthyosiform, erythroderma, and atrophic and ulcerative forms have been reported.^2^ Histopathologically, the typical features of sarcoidosis are naked granulomas with few inflammatory cells.^3^

## Case report

**Figure 1:**
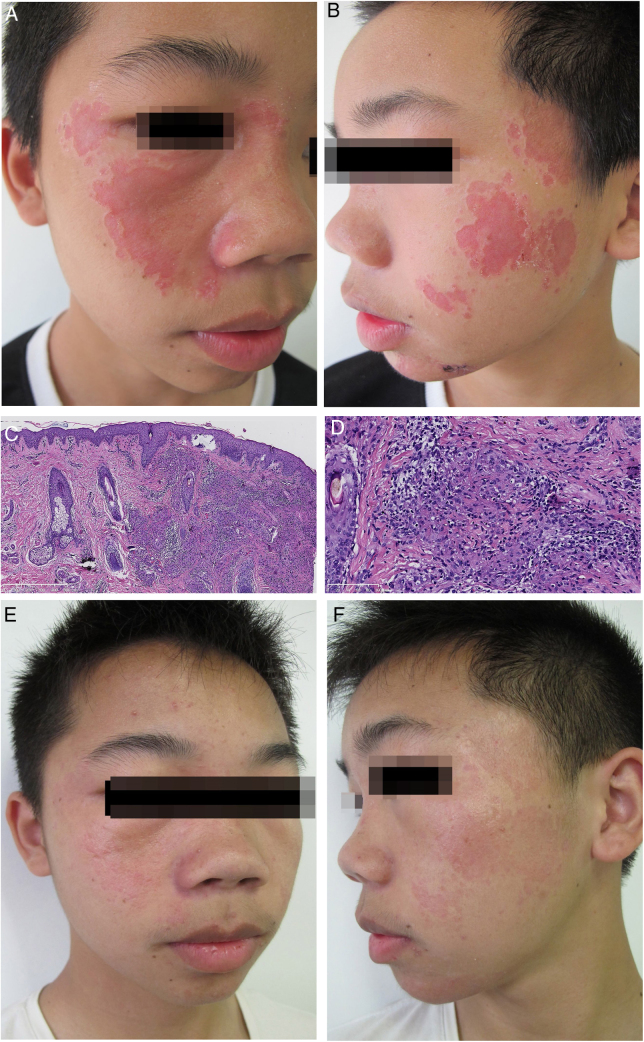
(A) Clinical presentation at the first visit. Multiple, well-defined, erythematous plaques involving the face. (B) The lesions are depressed with a cliff drop border. (C) Granulomatous reaction pattern characterized by multiple granulomas in the upper dermis (Haematoxylin & eosin, ×50). (D) Epithelioid cell granulomas, without central necrosis in association with a sparse lymphocytic infitrate (Haematoxylin & eosin, ×200). (E) Clinical presentation at the end of treatment, showing that most of the skin lesions disappeared. (F) With only a few reddish patches left.

A 13-year-old man presented with a 2-year history of multiple depressed skin lesions on his face. The lesions initially presented as asymptomatic erythematous patches which had gradually developed depressed centres over time. There was no history of trauma, ulceration, fever, cough, breathlessness, sensory loss, or intralesional steroid used to treat the lesions. Before presenting to our hospital, he had been diagnosed as lupus vulgaris in another hospital and had been given antituberculous therapy for 6 months. However, the skin lesions had gradually progressed during treatment. On physical examination, multiple erythematous plaques with a slightly atrophic appearance were seen on the patient's face (Fig. 1A and B). A full neurological examination was carried out, and the patient was found to have intact sensation. There was no palpable enlargement of peripheral nerves. Laboratory findings, including blood and urine routine examination, biochemistry investigations and antinuclear antibody test, were within normal limits. Purified protein derivative test proved to be negative, and a posterior-anterior chest X-ray revealed bilateral hilar lymphadenopathy. The skin biopsy showed dermal numerous compact epithelioid granulomas, surrounded by a collar of sparse lymphocytes with no evidence of acid-fast bacilli or fungi (Fig. 1C and D). There was no polarizable foreign material within the granulomas. We finally diagnosed the patient with atrophic cutaneous sarcoidosis. The patient was started on 0.5 mg/kg/day of prednisone (25 mg/day) and 6.0 mg/kg/day of hydroxychloroquine (300 mg/day). After 2 months, partially regression of the erythematous plaques and the bilateral hilar lymphadenopathy was achieved. Prednisone was tapered by 5 mg/month and was discontinued 4 months later. Then, oral administration of hydroxychloroquine was given for the consecutive 3 months. After 9 months of treatment, the patient presented an almost complete clinical response without bilateral hilar lymphadenopathy (Fig. 1E and F).

## Discussion

Cutaneous sarcoidosis with atrophic-appearing lesions is uncommon and has primarily been reported in association with ulcerative lesions, mostly on the lower legs of young and middle-aged people.^2^, ^4^ The facial atrophic form of sarcoidosis without associated ulceration in adolescents has rarely been described in the literature. In the absence of a definitive diagnostic test for sarcoidosis, most cases are diagnosed by histology and exclusion of other causes of granulomatous inflammation. Major differential diagnoses of the present case were: leprosy, systemic lupus erythematosus, and lupus vulgaris. Clinically, leprosy may spontaneously resolve, leaving atrophic skin. Diagnosis of leprosy is made by demonstration of acid-fast bacilli in skin biopsy or smear, clinical symptoms and neural involvement. The diagnosis of systemic lupus erythematosus could be ruled out because antinuclear antibodies were negative and there were no systemic symptoms. Ineffective antituberculous therapy could exclude lupus vulgaris. Histopathologically, sarcoidal granulomas can also be seen in multiple entities, including foreign body reactions, infections, and immunodeficiency syndromes.^5^ It is important to rule out infectious organisms and foreign substances. In our patient, the diagnosis of sarcoidosis was supported by the presence of hilar lymphadenopathy and histopathological findings. The rarity of cutaneous atrophic sarcoidosis makes it difficult to perform rigorous clinical trials and most of the information regarding treatments comes from case reports. The ulcerative-atrophic lesions tend to heal by using combination therapy with prednisone, hydroxychloroquine, and either mycophenolate mofetil or thalidomide.^4^ Although it had been reported that the atrophic form was resistant to treatment,^6^ our case responded well.

In conclusion, we diagnosed and curatively treated a patient with cutaneous atrophic sarcoidosis. In such patients, the long-course combination therapy with prednisone and hydroxychloroquine may achieve the clinical cure.

## Funding source

10.13039/501100003139CAMS Innovation Fund for Medical Sciences (CIFMS-2017-I2M-1-017).

## Authors' contributions

Xiaomei Zhu: Approval of the final version of the manuscript; elaboration and writing of the manuscript; obtaining, analysis, and interpretation of the data; effective participation in research orientation; critical review of the literature; critical review of the manuscript.

Jianfang Sun: Approval of the final version of the manuscript; critical review of the literature; critical review of the manuscript.

## Conflicts of interest

None declared.
